# Virtual Reality–Based Psychological Intervention for Young Adults Living With HIV: Protocol for a Randomized Controlled Trial

**DOI:** 10.2196/55633

**Published:** 2025-01-10

**Authors:** Hanxi Zhang, Jing Han, Ye Su, Hongxin Zhao, Fujie Zhang

**Affiliations:** 1 Beijing Ditan Hospital Capital Medical University Beijing China

**Keywords:** HIV, young adults, mental health, virtual reality, VR, psychological intervention, depression, anxiety, living with HIV, privacy, mental health, intervention

## Abstract

**Background:**

Young adults (15-24 years old) living with HIV may experience pressure both from HIV infection and social role change problems, resulting in a series of psychological problems such as depression and anxiety. Effective psychological intervention can improve their mental health and quality of life.

**Objective:**

The study aims to explore the effectiveness of VR-based mental intervention on young adults living with HIV. The application and advantages of virtual reality (VR) in children’s psychotherapy provide new ideas for psychological intervention for young adults living with HIV.

**Methods:**

We use the qualitative interviews and questionnaire results as well as guided by classical psychotherapy to create a personalized psychological intervention system for young adults living with HIV through VR technology, which is based on the long-term AIDS treatment cohort and infectious diseases cohort of children. We use the mental scales and biochemical indexes as the outcomes, conducting a prospective randomized controlled trial to verify the feasibility and effectiveness of the VR psychological intervention system.

**Results:**

The study began enrollment in September 2023. To date, 160 participants have finished the baseline questionnaires.

**Conclusions:**

The study results might provide a scientific basis for accurate psychological treatment among young adults living with HIV in the future.

**International Registered Report Identifier (IRRID):**

DERR1-10.2196/55633

## Introduction

According to the latest data from the joint United Nations Program on HIV/AIDS (UNAIDS), an average of about 4000 people were newly diagnosed with HIV globally per day in 2021. Young people aged 15-24 years old accounted for 31% of new HIV infections. In the Asia-Pacific region, about 260,000 young people were newly diagnosed with HIV in 2021, accounting for 17.3% of the global increase, second only to Africa [1]. The mental health of people living with HIV/AIDS is an important part of the quality of life, and infection-related stigma makes people living with HIV/AIDS face 2-3 times more anxiety and depression than the general population [2-4]. Young people living with HIV face pressure from social adaptation, such as personal growth and role change, as well as disease pressure caused by the incurability and stigma of HIV infection [5]. Studies on young people aged 10-24 years old living with HIV showed that 21% of them had depression symptoms and 14% had anxiety, and the proportion interval of suicidal ideation was from 4.4% to 24.4%, and those with long-term suicidal ideation ranged from 9.26% to 33.33% [6-9]. So, how to carry out effective psychological intervention for young people living with HIV is very important.

There are many forms of psychological intervention, and numerous interventions are intended to support mental health, mainly by way of face-to-face and group-based intervention, internet intervention, and mobile remote intervention. Regardless of the form of psychological intervention, it is based on the theoretical framework or theoretical basis. Common theoretical bases include cognitive behavioral therapy (CBT) and dialectical behavior therapy (DBT) [10]. Previous studies proved the effectiveness of CBT or DBT in the way of face-to-face intervention. Synder et al [11] conducted a group-based psychological intervention study on people living with HIV aged between 16 and 24 years of CBT. The results showed that the self-perceived social support of the intervention group was higher than that of the control group, but the difference was not statistically significant (*P*=.20) [11]. Bedics et al [12] conducted a 1-year DBT treatment on 101 women with borderline personality disorder, which showed that DBT therapy could reduce suicidal behaviors and nonsuicidal self-injury behaviors. Burton et al [13] conducted a psychological intervention with DBT therapy, suggesting that the depression clinical scores had improved of the participants in the intervention group. DBT therapy has achieved some effects in helping improve behavioral problems in children [14] and alcohol problems among college students [15]. However, face-to-face and group-based CBT or DBT needs to be carried out by professionals, and the existing qualified health care providers and infrastructures are limited in meeting the challenge in China.

Studies in developed countries have shown the feasibility and efficacy of using mHealth to provide counseling and services to improve mental health in diverse populations. Mohr et al [16] conducted a self-control psychological intervention in the general population by mobile phone, and the results showed that depression and anxiety decreased over time [16]. The research team conducted a randomized controlled psychological intervention trial on 300 adults living with HIV on the WeChat public platform. At 3 and 6 months after the intervention, the depression level and suicide ideation rate of the experimental group were reduced compared with the control group (*P*=.02) [17,18]. However, there is no study focusing on mental intervention among young people living with HIV. At the same time, studies showed that compliance with remote Internet or mobile phone intervention is poor, and patients are unable to concentrate during the intervention process, which makes it difficult to ensure the effective implementation of the intervention.

Virtual reality (VR) provides a variety of sensory stimuli to people through the computer-generated virtual environment, enabling people to interact with the virtual environment naturally to achieve a sense of immersion and experience in the real environment [19]. Falconer et al [20] used VR technology to set virtual scenes to treat patients with depression and found that the depression decreased after the intervention, and the improvement effect was still maintained one month after the end of the experiment. Based on the mindfulness-based program (MBP), Modrego-Alarcón et al [21] conducted a study on the stress level of 280 college students through VR equipment. The results showed that the experimental group had increased relaxation and decreased stress levels compared with the control group (*P*=.006) [21]. Navarro-Haro et al [22] combined DBT therapy with VR to guide mindfulness training. The results showed that the impulse to commit suicide, self-harm, give up treatment, and use drugs in the experimental group was reduced after the intervention [22]. However, most of the existing studies are small-scale pilot studies on general adults or adolescents with adverse psychological conditions, and there is little study on young people living with HIV.

In summary, the proposed study intends to address the issues of prevalent mental health problems in young people living with HIV and to innovatively use VR to provide integrated and culturally appropriate intervention for mental health improvement.

## Methods

### Goal and Objectives

The objectives of the project are (1) using DBT as the theoretical framework, based on VR technology to carry out the individualized psychological intervention for young people living with HIV; and (2) A 1:1 prospective randomized controlled trial (RCT) will be conducted to verify the effectiveness of VR psychological intervention on reducing depression and anxiety in young people living with HIV.

### Intervention Design

#### Development of A VR Intervention System

##### Content of the Intervention

The proposed study is guided by DBT and combined with the actual needs and unique characteristics of young people living with HIV to develop a specialized psychological intervention system suitable for the characteristics of this population.

The actual needs of mental health among young people living with HIV were collected based on literature and qualitative interviews with young people living with HIV. The outline of the qualitative interview mainly included (1) basic information and (2) The main source of stress (infection, drug side-effect, income, life expectancy).

**Figure 1:**
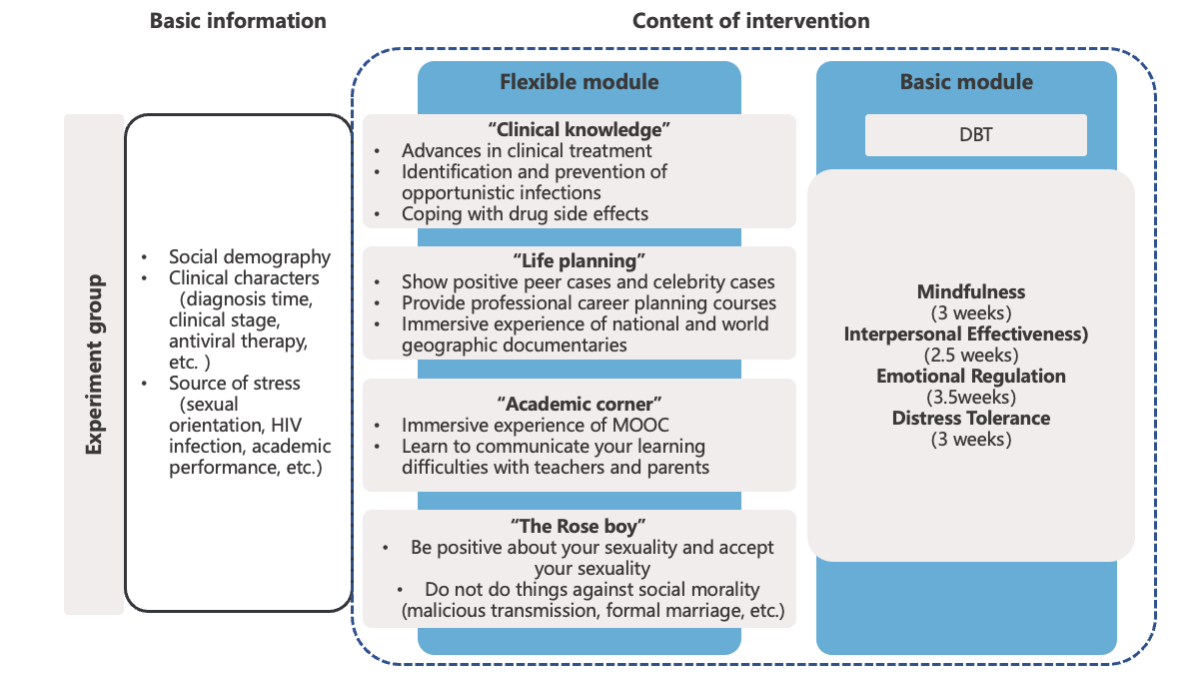
Psychological intervention content in the experimental group. MOOC: Massive Open Online Courses. DBT: dialectical behavior therapy.

Study subjects in the experimental group received 2 modules of psychological intervention. Module 1 was the basic intervention module, mainly the DBT treatment course, which covers 4 parts: mindfulness, distress tolerance, emotional regulation, and interpersonal effectiveness. The second module is a flexible intervention module applicable to the actual needs of young people living with HIV. As shown in Figure 1.

##### Intervention Delivery

We set up a user management platform as the support for the intervention. The function of the platform included (1) information management: a complete record with a unique number is generated when each research object is enrolled. The researcher is the system administrator and can add and modify the relevant information of the patient, such as fixed number, basic personal information (deprivatized), clinical and therapeutic information, psychological assessment scale, etc. The system is associated with the VR intervention software, which can record and synchronize the completion of the intervention course of the research object, and the administrator can view and export the corresponding data through the number.

(2) Upload and update intervention resources: the intervention content involved in VR software forms a “resource library” in the management system, and the user portrait is generated when the subject is enrolled in the corresponding intervention content. The user portrait is the virtualization of real users and the category of research objects extracted; that is, the entire psychological intervention system is a big picture. Against this background, different types of subjects receive psychological intervention corresponding to their characteristics to achieve personalized customization. In the course of the experiment, the researchers continuously improved and updated the intervention resources.

(3) The intervention goal setting and process supervision: researchers use motivation type interview (motivational interviewing [MI]) to help subjects set up goals, regularly to supervise the completion of the intervention for subjects, and set a message to remind, intervention to ensure compliance.

##### VR Application Development

**Figure 2:**
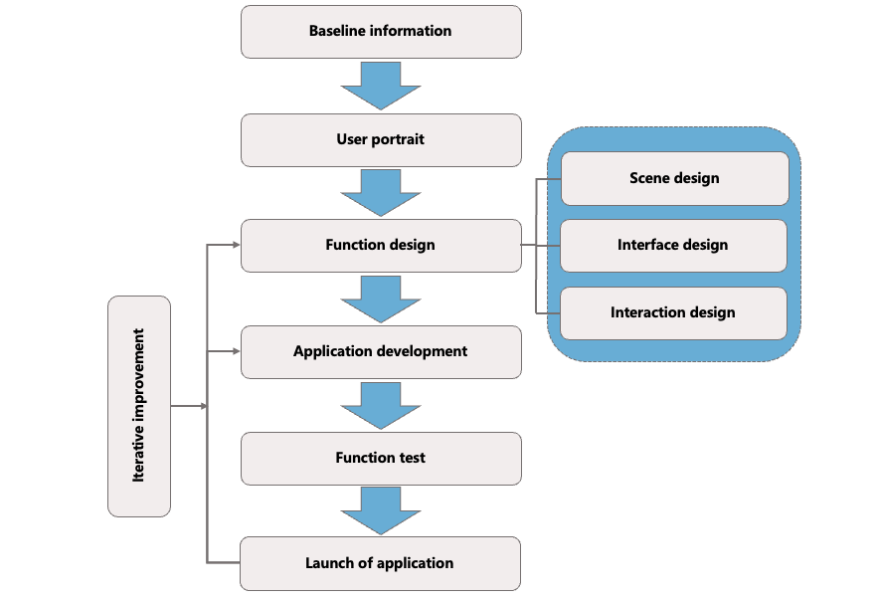
Virtual reality (VR) system development.

VR application development mainly includes 3 stages. First, Scene design is a virtual scene in which the participants experience psychological intervention therapy. This study mainly includes 2 scenes: natural landscape and indoor landscape. The natural landscape was used as the environment for participants to immerse in mindfulness and other skills training. The indoor landscape, including the home environment and the consultation environment, was used for the explanation and learning of psychological intervention courses. Second, regarding interface design, this study follows the principle of consistency of interface structure and style, choosing simple graphical design language and interface style with comfort and color sense. The icon uses a simple 2-dimensional graphic simulation to represent the functional form, which is convenient for the participant to identify and quickly associate the icon function, reducing the possibility of wrong selection. Third, interaction design refers to the use of VR hardware devices such as glasses and gamepads to realize the interaction of subjects in the scene. For example, when the participant enters the “family scene,” he or she can move freely and roam in panorama; through the button control of indoor lighting and curtains, choose the bedroom, living room, consulting room, etc, to carry out the study of psychological courses. As shown in Figure 2.

##### Verify the Effectiveness of VR Psychological Intervention

**Figure 3:**
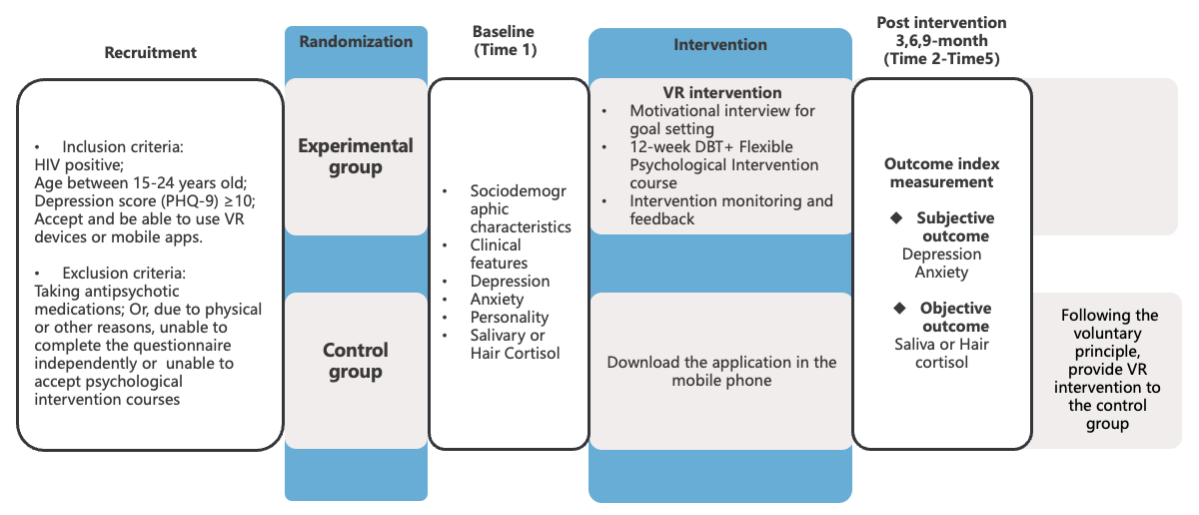
The process of the intervention. PHQ-9: Patient Health Questionnaire-9. VR: virtual reality. DBT: dialectical behavior therapy.

A 1:1 RCT will be conducted to verify the effectiveness of VR psychological intervention in reducing depression and anxiety in young people living with HIV. The RCT was not possible to blind the participants. The study lasted for 12 months. According to the inclusion and exclusion criteria, the participants are randomly divided into an experimental group and a control group. The experimental group received VR psychological intervention for 3 months, and the control group received non-VR mobile phone intervention for the same period. Data will be collected at 5 time points: baseline, postintervention, 3 months, 6 months, and 9 months after intervention, as shown in Figure 3.

##### Measurement

Depression score was measured by the Patient Health Questionnaire-9 (PHQ-9). PHQ-9 is shorter but has demonstrated both good reliability and validity in the Chinese populations [23,24]. The scale consists of 9 items, such as “Little interest or pleasure in doing things” and “Feeling down, depressed, or hopeless,” to measure the depressive status of the participants over the previous 2 weeks. Each item was rated on a 4-point Likert scale ranging from 0 (not at all) to 3 (nearly every day), providing a 0 to 27 total severity score, with higher scores indicating increased depressive symptoms [25].

Anxiety was measured by the Self-rating Anxiety Scale (SAS) that assesses anxiety in the last week with the Chinese version [26,27]. SAS has 20 items with responses of a 4-point Likert scale (1=“never or rarely,” 2=“sometimes,” 3=“most of the time,” 4=“almost every day”). The total score ranges from 20-80, with 50 as a cutoff point, 50-59 being considered mild anxiety, 60-69 as moderate anxiety, and ≥70 as severe anxiety.

##### Study Participants

Inclusion criteria were a positive HIV test, age 15-24 years, depression score (PHQ-9)≥10 (with depressive symptoms), accepting and being able to use VR devices or mobile apps. Exclusion criteria were taking antipsychotic drugs or, for physical or other reasons, being unable to complete surveys or questionnaires independently, or being unable to accept psychological intervention courses.

##### Recruitment

The recruitment location is mainly based on Beijing Ditan Hospital Capital Medical University, which is treating 11,735 people in 2023. After clinic doctors have completed all the treatment procedures, researchers will distribute research leaflets to them to introduce the research project and invite them to participate. If the young people living with HIV agree to participate, the program’s researchers will invite the participants to a separate room to be screened for enrollment conditions.

The staff will present the informed consent form of this study and inform the content of the informed consent form, including the introduction of the project, the benefits and possible risks of participating in this study, and the voluntary principle. The research subjects will be asked to sign the informed consent form after they have fully understood it.

##### Baseline Information Collection

The collection of baseline information in this study included 2 items: filling out questionnaires and retaining biological samples. A self-designed questionnaire was used to collect baseline information, including sociodemographic characteristics, medication and treatment information, clinical indicators, and mental health scale evaluation. The electronic questionnaire data can be downloaded directly, which is convenient to timely grasp or correct the information of infected people enrolled on the same day, and ensure the quality of data. To facilitate data collection, improve work efficiency, and improve data collection quality, electronic questionnaires were used at baseline and 3 follow-up visits.

##### Sample Size Calculation and Group Randomization

The sample size was calculated according to depression, the main outcome indicator of this study. Participants with PHQ-9 depression scale score ≥10 were classified as having depressive symptoms. It is expected that after intervention, the proportion of depression in the intervention group will be reduced by 20% compared with that in the control group. A bilateral test with a sample size ratio of the test group and control group as 1:1, test efficacy (1-β) as 0.9, and α as .05 was adopted, and a loss rate of 20% is expected. Through the calculation of PASS software (NCSS), 145 study subjects need to be included, and finally, 160 study subjects are rounded up, including 80 in the experimental group and 80 in the control group.

To ensure the balance between the experimental group and the control group, the block randomization method was adopted, code was written in R (R Foundation for Statistical Computing), random numbers were generated according to random seeds, and randomization was carried out to make the allocation of each treatment group more balanced and meet the research requirements. According to the order in which the subjects were enrolled, each participant was given a random number generated by the above random scheme. According to the group corresponding to the random number, the subjects were randomly assigned to the experimental group and the control group.

##### Quality Control

To improve the compliance of young people living with HIV, this study conducted regular telephone communication with the permission of the study subjects based on unified management through the user management platform to understand the intervention and treatment of the study subjects and give more humanistic care.

To ensure privacy and reduce reporting bias, this study was conducted anonymously, and the VR intervention was free during the study. The fixed and unique number assigned to each research subject when they were enrolled was used as the basis for information matching. After each collection of questionnaires, data was downloaded and backed up on the same day, and the questionnaire platform data was deleted in time. The downloaded questionnaires were formed into a special database and stored encrypted in an unconnected computer for safekeeping by the project leader. At the same time, the VR intervention software and the intervention program of the control group are regularly maintained, network security is regularly checked, user information is protected in many aspects, and security information is set in the application, such as adding mobile phone number association, email association, secret security issues, and the relevant computer professionals are supervised.

##### Statistical Analysis

Quantitative data were analyzed using SD or demographic characteristics of composition comparison baseline. The *t* test, ANOVA, or rank sum test were used for hypothesis testing of quantitative data. The chi-square test or Fisher exact probability method was used to test the hypothesis of qualitative data. The effect size would also be calculated. In this study, the intervention effect of the VR psychological intervention system was analyzed using generalized estimating equations (GEE). During the follow-up, the attrition diagram in each group would be plotted over time. In this study, binary classification PHQ-9 is used as the outcome variable, which belongs to longitudinal data, and there is a certain correlation between the data. When conducting statistical analysis of such data, the data correlation should be fully considered. Therefore, GEE was used to analyze the intervention effect to correct for the correlation between data caused by repeated measurements at multiple time points.

##### Study Significance

The clinical cohort supported by this study is a long-term antiviral treatment cohort, which can ensure the sample size and representativeness of the enrolled participants. The research team includes experts and scholars from various disciplines, including epidemiology and health statistics, clinical medicine, psychology, computer science, technology, etc, to provide professional guidance for the design of a psychological intervention department and the implementation of mental health intervention. The case managers in the research team are from Beijing Red Ribbon Home and have rich experience in providing psychological support for people living with HIV. The Home of Red Ribbon was previously known as the Home of Red Ribbon of Beijing Ditan Hospital, which was established in 1999. On January 5, 2005, the Home of Red Ribbon was registered with the Beijing Municipal Civil Affairs Bureau as the first civil society organization in China specializing in comprehensive HIV/AIDS care. It has 6 independent branches focusing on medical support, self-help of HIV-positive people, volunteer service, social assistance, online publicity, and legal aid. Its purpose is to provide comprehensive support and services for people living with HIV by pooling strength from all social strata, carrying out a variety of HIV prevention and control measures, and health awareness campaigns for enhanced public understanding of HIV.

This study innovatively applied modern VR technology to psychological intervention for young people living with HIV, which provides a new idea for our country to carry out psychological treatment. As a special group, people living with HIV are in urgent need of effective psychological guidance and sensitivity for privacy. This study combines VR technology with psychological intervention, uses an immersive 3D virtual environment to overcome the disadvantages of 2D mobile phone intervention, and avoids the privacy exposure that may be caused by offline medical treatment. It can provide data support and a scientific basis for solving the mental health problems of young people living with HIV in the future.

### Ethical Considerations

This study was part of a research project that had been approved by the ethics committee of Beijing Ditan Hospital of Capital Medical University (Approval number: 2024-012). All individual participants were provided oral informed consent at the time of enrolment.

## Results

This study was funded by the Capital Medical University Research Development Fund (grant PYZ22138) in April 2023. The enrollment started in September 2023. The data analysis is expected to be finished in April 2025.

## Discussion

This study innovatively transcends traditional intervention models to conduct a prospective randomized controlled trial of VR-based psychological intervention for young people living with HIV in China. The research results will provide the effectiveness of VR-based mental intervention among young people living with HIV.

This research might provide yields practical data that advocates for the integration of mental health services into the standardized treatment model for HIV, as well as the wider implementation of psychological intervention services within domestic HIV-designated medical institutions and other health care facilities lacking specialized psychological consultation clinics.

The limitation of the study was that the population involved in this study mainly were young people living with HIV, which might cause a selection bias. However, young HIV-positive people were a group with a high proportion of HIV prevalence in China, so the study results still were representative of the effectiveness of the VR intervention and had good prospects of the intervention expanding in China.

The study population needs to be expanded to include people with chronic diseases to achieve effective mental intervention based on VR technology in the future.

## Abbreviations

CBT: cognitive behavioral therapy
DBT: dialectical behavior therapy
GEE: generalized estimating equations
MBP: mindfulness-based program
MI: motivational interviewing
PHQ-9: Patient Health Questionnaire-9
RCT: randomized controlled trial
SAS: Self-rating Anxiety Scale
UNAIDS: United Nations Program on HIV/AIDS
VR: virtual reality

## Data Availability

The datasets generated during and/or analyzed during this study are available from the corresponding author on reasonable request.

## References

[1] UNAIDS UNAIDS data 2021.

[2] Vreeman RC, McCoy BM, Lee S (2017). Mental health challenges among adolescents living with HIV. J Int AIDS Soc.

[3] Shenderovich Y, Boyes M, Esposti MD, Casale M, Toska E, Roberts KJ, Cluver L (2021). Relationships with caregivers and mental health outcomes among adolescents living with HIV: a prospective cohort study in South Africa. BMC Public Health.

[4] Rubin LH, Maki PM (2019). HIV, depression, and cognitive impairment in the era of effective antiretroviral therapy. Curr HIV/AIDS Rep.

[5] Shang X (2021). Development of social adjustment scale for adolescents infected with HIV. Peking Union Medical College. 2021. (In Chinese).

[6] Brown LK, Whiteley L, Harper GW, Nichols S, Nieves A, ATN 086 Protocol Team for The Adolescent Medicine Trials Network for HIV/AIDS Interventions (2015). Psychological symptoms among 2032 youth living with HIV: a multisite study. AIDS Patient Care STDS.

[7] Casale M, Boyes M, Pantelic M, Toska E, Cluver L (2019). Suicidal thoughts and behaviour among South African adolescents living with HIV: can social support buffer the impact of stigma?. J Affect Disord.

[8] Adeyemo S, Adeosun II, Ogun OC, Adewuya A, David AN, Adegbohun AA, Adejumo O, Ogunlowo OA, Adeyemo OO (2020). Depression and suicidality among adolescents living with human immunodeficiency virus in Lagos, Nigeria. Child Adolesc Psychiatry Ment Health.

[9] Lee B, Chhabra M, Oberdorfer P (2011). Depression among vertically HIV-infected adolescents in Northern Thailand. J Int Assoc Physicians AIDS Care (Chic).

[10] Lin X (2021). The effect of dialectical behavior therapy on adolescent depression with non-suicidal self-injury. Chongqing Medical University.2021,05. (In Chinese).

[11] Snyder K, Wallace M, Duby Z, Aquino LDH, Stafford S, Hosek S, Futterman D, Bekker LG (2014). Preliminary results from hlanganani (coming together): a structured support group for HIV-infected adolescents piloted in Cape Town, South Africa. Child Youth Serv Rev.

[12] Bedics JD, Atkins DC, Harned MS, Linehan MM (2015). The therapeutic alliance as a predictor of outcome in dialectical behavior therapy versus nonbehavioral psychotherapy by experts for borderline personality disorder. Psychotherapy (Chic).

[13] Burton ET, Kamody RC, Pluhar EI, Gray E, Abdullah S (2020). Radical acceptance and obesity-related health conditions: a case report. J Clin Psychol Med Settings.

[14] Perepletchikova F, Kaufman J (2010). Emotional and behavioral sequelae of childhood maltreatment. Curr Opin Pediatr.

[15] Whiteside U, Cronce JM, Pedersen ER, Larimer ME (2010). Brief motivational feedback for college students and adolescents: a harm reduction approach. J Clin Psychol.

[16] Mohr DC, Tomasino KN, Lattie EG, Palac HL, Kwasny MJ, Weingardt K, Karr CJ, Kaiser SM, Rossom RC, Bardsley LR, Caccamo L, Stiles-Shields C, Schueller SM (2017). IntelliCare: an eclectic, skills-based app suite for the treatment of depression and anxiety. J Med Internet Res.

[17] Zhang H (2018). Study on the effect of psychological intervention on HIV /AIDS patients with mobile health (mHealth). Sun Yat-sen Universit. (In Chinese).

[18] Li Y, Guo Y, Hong YA, Zhu M, Zeng C, Qiao J, Xu Z, Zhang H, Zeng Y, Cai W, Li L, Liu C (2019). Mechanisms and effects of a WeChat-based intervention on suicide among people living with HIV and depression: path model analysis of a randomized controlled trial. J Med Internet Res.

[19] Zhu L, Guo L, Li Y (2021). Application and prospect of virtual reality technology in healthcare field. Information and computers. . (In Chinese).

[20] Falconer CJ, Rovira A, King JA, Gilbert P, Antley A, Fearon P, Ralph N, Slater M, Brewin CR (2016). Embodying self-compassion within virtual reality and its effects on patients with depression. BJPsych Open.

[21] Modrego-Alarcón M, López-Del-Hoyo Y, García-Campayo J, Pérez-Aranda A, Navarro-Gil M, Beltrán-Ruiz M, Morillo H, Delgado-Suarez I, Oliván-Arévalo R, Montero-Marin J (2021). Efficacy of a mindfulness-based programme with and without virtual reality support to reduce stress in university students: a randomized controlled trial. Behav Res Ther.

[22] Navarro-Haro MV, López-Del-Hoyo Y, Campos D, Linehan MM, Hoffman HG, García-Palacios A, Modrego-Alarcón M, Borao L, García-Campayo J (2017). Meditation experts try virtual reality mindfulness: a pilot study evaluation of the feasibility and acceptability of virtual reality to facilitate mindfulness practice in people attending a mindfulness conference. PLoS One.

[23] Sun XY, Li YX, Yu CQ, Li LM (2017). [Reliability and validity of depression scales of Chinese version: a systematic review]. Zhonghua Liu Xing Bing Xue Za Zhi.

[24] Chen S, Fang Y, Chiu H, Fan H, Jin T, Conwell Y (2013). Validation of the nine-item patient health questionnaire to screen for major depression in a Chinese primary care population. Asia Pac Psychiatry.

[25] Kroenke K, Spitzer RL, Williams JB (2001). The PHQ-9: validity of a brief depression severity measure. J Gen Intern Med.

[26] Li H, Jin D, Qiao F, Chen J, Gong J (2016). Relationship between the self-rating anxiety scale score and the success rate of 64-slice computed tomography coronary angiography. Int J Psychiatry Med.

[27] Zhang Y, Liu R, Li G, Mao S, Yuan Y (2015). The reliability and validity of a Chinese-version short health anxiety inventory: an investigation of university students. Neuropsychiatr Dis Treat.

